# 9-(Thio­phen-2-yl)-8,9-dihydro-3*H*-pyrazolo­[4,3-*f*]quinolin-7(6*H*)-one ethanol monosolvate

**DOI:** 10.1107/S1600536812033533

**Published:** 2012-08-01

**Authors:** Juhua Peng, Runhong Jia

**Affiliations:** a Lianyungang Teacher’s College, Lianyungang 222006, People’s Republic of China

## Abstract

In the title compound, C_14_H_11_N_3_OS·C_2_H_5_OH, the dihedral angle between the pyridine N—C_fused_—C_fused_—C(thio­phene) plane and the plane of the thio­phene ring is 81.9 (3)°, indicating that they are close to perpendicular. The dihedral angle between this pyridine plane and the benzene ring is 1.3 (3)°. The thio­phene ring is disordered over two coplanar orientations with an occupancy ratio of 0.692 (7):0.308 (7), while the ethanol solvent mol­ecule is also disordered over two sets of site in a 0.66 (4):0.34 (4) ratio. In the crystal, chains are formed along the *b* axis by N—H⋯O and O—H⋯N inter­actions with adjacent chains being connected through C—H⋯N and C—H⋯S inter­actions.

## Related literature

For background to the biological activity of quinolinone derivatives, see: Larsen *et al.* (1996 ▶); Chackal *et al.* (2002 ▶); Kalluraya & Sreenivasa (1998 ▶); Xu *et al.* (2000 ▶). For the synthesis of quinolino­nes, see: Suarez *et al.* (1999 ▶). 
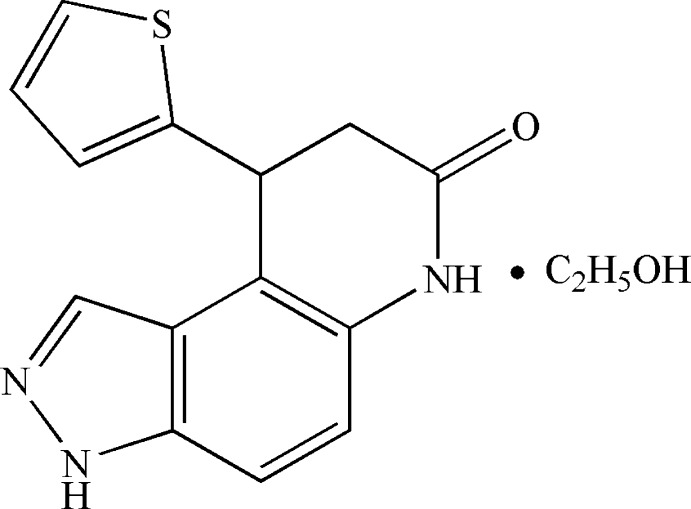


## Experimental

### 

#### Crystal data

C_14_H_11_N_3_OS·C_2_H_6_O
*M* *_r_* = 315.39
Monoclinic, 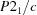

*a* = 9.3831 (10) Å
*b* = 19.138 (2) Å
*c* = 8.7490 (9) Å
β = 99.412 (1)°
*V* = 1549.9 (3) Å^3^
*Z* = 4
Mo *K*α radiation
μ = 0.22 mm^−1^
*T* = 298 K
0.38 × 0.19 × 0.12 mm

#### Data collection

Bruker SMART CCD area-detector diffractometer
Absorption correction: multi-scan (*SADABS*; Sheldrick, 1996 ▶) *T*_min_ = 0.921, *T*_max_ = 0.974
7663 measured reflections
2707 independent reflections
1526 reflections with *I* > 2σ(*I*)
*R*_int_ = 0.041

#### Refinement

*R*[*F* ^2^ > 2σ(*F* ^2^)] = 0.052
*wR*(*F* ^2^) = 0.154
*S* = 1.02
2707 reflections
248 parameters
?
Δρ_max_ = 0.28 e Å^−3^
Δρ_min_ = −0.26 e Å^−3^

### 

Data collection: *SMART* (Bruker, 1998 ▶); cell refinement: *SAINT* (Bruker, 1999 ▶); data reduction: *SAINT*; program(s) used to solve structure: *SHELXS97* (Sheldrick, 2008 ▶); program(s) used to refine structure: *SHELXL97* (Sheldrick, 2008 ▶); molecular graphics: *SHELXTL* (Sheldrick, 2008 ▶); software used to prepare material for publication: *SHELXTL*.

## Supplementary Material

Crystal structure: contains datablock(s) global, I. DOI: 10.1107/S1600536812033533/zq2174sup1.cif

Structure factors: contains datablock(s) I. DOI: 10.1107/S1600536812033533/zq2174Isup2.hkl

Supplementary material file. DOI: 10.1107/S1600536812033533/zq2174Isup3.cml

Additional supplementary materials:  crystallographic information; 3D view; checkCIF report

## Figures and Tables

**Table 1:** Hydrogen-bond geometry (Å, °)

*D*—H⋯*A*	*D*—H	H⋯*A*	*D*⋯*A*	*D*—H⋯*A*
N1—H1⋯O2^i^	0.86	1.99	2.838 (16)	170
N3—H3⋯O1^ii^	0.86	2.04	2.863 (4)	160
O2—H2⋯N2	0.82	2.05	2.855 (14)	167
C8—H8⋯S1^iii^	0.98	2.86	3.802 (6)	162
C9—H9*A*⋯N1^iii^	0.97	2.56	3.529 (7)	175

Symmetry codes: (i) 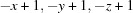; (ii) 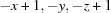; (iii) 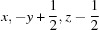.

**Fig. 1.**
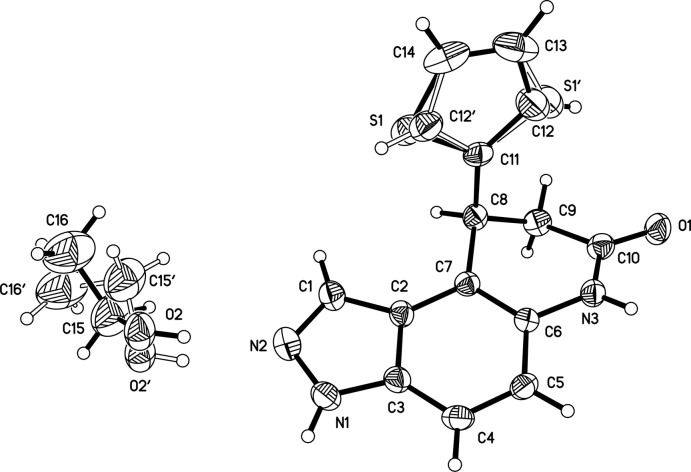
The molecular structure of title compound, showing 30% probability displacement ellipsoids.

**Fig. 2.**
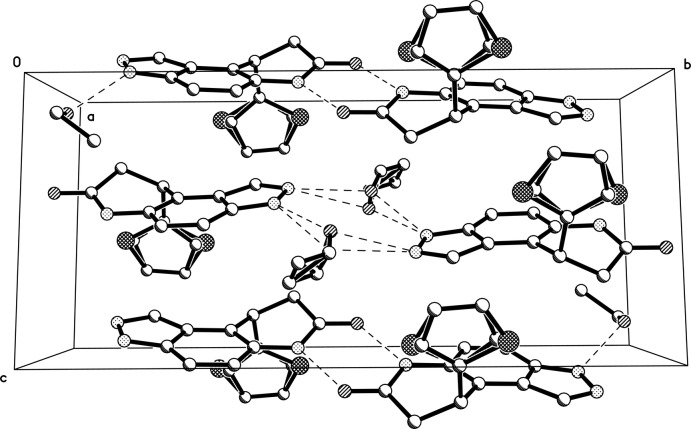
A packing diagram of title compound viewed along the a axis.

## supplementary crystallographic information

### Comment

The quinoline ring system is an important structural unit widely existing in alkaloids, therapeutics and synthetic analogues with interesting biological activities (Larsen *et al.*, 1996). A large variety of quinoline derivatives have been used as antimalarial, anti-inflammatory, antiasthmatic, antibacterial, antihypertensive and tyrokinase PDGF-RTK inhibiting agents (Kalluraya & Sreenivasa, 1998). Various quinolinone derivatives are known to display interesting biological properties, for example, quinolinones represent the structural basis of many biologically active compounds, such as those with cardiovascular, anti-osteoporosis, anti-tumor (Chackal *et al.*, 2002), antiinflammatory, and anti-virus (Xu *et al.*, 2000) activities and so on.

Due to their diverse ranges of biological properties, the synthesis of these important molecules has attracted widespread attention. Some researchers have reported the synthesis of quinolinones (Suarez *et al.*, 1999). To the best of our knowledge, however, the pyrazolo[4,3-*f*]quinolin-7-one derivatives have not been investigated. Because of the biological activities they exhibit, these compounds have distinguished themselves as heterocycles of profound chemical and biological significance.

In this paper we report the crystal structure of the title compound, C_14_H_11_N_3_O_S_.C_2_H_6_O, which was synthesized by the reaction of thiophene-2-carbaldehyde, 2,2-dimethyl-1,3-dioxane-4,6-dione, and indazol-5-amine in ethylene glycol without catalyst under microwave irradiation.

In the crystal structure of the title compound, the pyridine ring exhibits an envelope-like structure. The dihedral angle between the pyridine C6/C7/C8/N3 plane and the C11/C12/C13/C14/S1 thiophene ring is 81.9 (3)°, indicating that they are close to perpendicular. The dihedral angle between the pyridine C6/C7/C8/N3 plane and the C2—C7 benzene ring is 1.3 (3)°. The thiophene ring is disordered over two coplanar orientations with an occupancy ratio of 0.692 (7):0.308 (7) while the ethanol solvent molecule is also disordered over two sets of positions with a ratio of 0.66 (4):0.34 (4). Chains are formed along the *b* axis by N-H···O and O-H···N interactions and adjacent chains are connected through C-H···N and C-H···S interactions.

### Experimental

The title compound was prepared by the reaction of thiophene-2-carbaldehyde (1 mmol), 2,2-dimethyl-1,3-dioxane-4,6-dione (1 mmol), and indazol-5-amine (1 mmol) in ethylene glycol (1.0 ml). Single crystals were obtained by slow evaporation of a 95% aqueous ethanol solution (yield 70%; m.p. 553–554 K).

IR (cm^-1^): 3194, 3013, 2967, 1681, 1502, 1390, 1241, 1162, 1049, 937, 843, 704. ^1^H NMR (DMSO-d_6_): 13.03 (s, 1H, NH), 10.21 (s, 1H, NH), 7.42 (d, J = 8.8 Hz, 1H, ArH), 7.31–7.30 (m, 1H, ArH), 7.02 (d, J = 8.8 Hz, 1H, ArH), 6.92–6.87 (m, 2H, ArH), 4.98 (d, J = 4.4 Hz, 1H, CH), 3.12–3.06 (m, 1H, CH_2_), 2.77–2.72 (m, 1H, CH_2_).

### Refinement

All H atoms were positioned geometrically and treated as riding, with N—H = 0.86 Å and *U*_iso_(H) = 1.2*U*_eq_(N), with C—H = 0.93 Å and *U*_iso_(H) = 1.2*U*_eq_(C) for aromatic H atoms, with C—H = 0.97 Å and *U*_iso_(H) = 1.2*U*_eq_(C) for methylene H atoms, with C—H = 0.96 Å and *U*_iso_(H) = 1.5*U*_eq_(C) for methyl H atoms, and with O—H = 0.82 Å and *U*_iso_(H) = 1.5*U*_eq_(O).

### Crystal data

**Table:**

C_14_H_11_N_3_OS·C_2_H_6_O	*F*(000) = 664
*M**_r_* = 315.39	*D*_x_ = 1.352 Mg m^−^^3^
Monoclinic, *P*2_1_/*c*	Melting point = 553–554 K
Hall symbol: -P 2ybc	Mo *K*α radiation, λ = 0.71073 Å
*a* = 9.3831 (10) Å	Cell parameters from 1612 reflections
*b* = 19.138 (2) Å	θ = 2.4–25.1°
*c* = 8.7490 (9) Å	µ = 0.22 mm^−^^1^
β = 99.412 (1)°	*T* = 298 K
*V* = 1549.9 (3) Å^3^	Block, colourless
*Z* = 4	0.38 × 0.19 × 0.12 mm

### Data collection

**Table:**

Bruker SMART CCD area-detector diffractometer	2707 independent reflections
Radiation source: fine-focus sealed tube	1526 reflections with *I* > 2σ(*I*)
Graphite monochromator	*R*_int_ = 0.041
phi and ω scans	θ_max_ = 25.0°, θ_min_ = 2.1°
Absorption correction: multi-scan (*SADABS*; Sheldrick, 1996)	*h* = −11→8
*T*_min_ = 0.921, *T*_max_ = 0.974	*k* = −19→22
7663 measured reflections	*l* = −10→10

### Refinement

**Table:**

Refinement on *F*^2^	Primary atom site location: structure-invariant direct methods
Least-squares matrix: full	Secondary atom site location: difference Fourier map
*R*[*F*^2^ > 2σ(*F*^2^)] = 0.052	Hydrogen site location: inferred from neighbouring sites
*wR*(*F*^2^) = 0.154	*w* = 1/[σ^2^(*F*_o_^2^) + (0.0595*P*)^2^ + 0.7752*P*] where *P* = (*F*_o_^2^ + 2*F*_c_^2^)/3
*S* = 1.02	(Δ/σ)_max_ = 0.001
2707 reflections	Δρ_max_ = 0.28 e Å^−^^3^
248 parameters	Δρ_min_ = −0.26 e Å^−^^3^
0 restraints	

### Special details

**Table:**

Geometry. All e.s.d.'s (except the e.s.d. in the dihedral angle between two l.s. planes) are estimated using the full covariance matrix. The cell e.s.d.'s are taken into account individually in the estimation of e.s.d.'s in distances, angles and torsion angles; correlations between e.s.d.'s in cell parameters are only used when they are defined by crystal symmetry. An approximate (isotropic) treatment of cell e.s.d.'s is used for estimating e.s.d.'s involving l.s. planes.
Refinement. Refinement of *F*^2^ against ALL reflections. The weighted *R*-factor *wR* and goodness of fit *S* are based on *F*^2^, conventional *R*-factors *R* are based on *F*, with *F* set to zero for negative *F*^2^. The threshold expression of *F*^2^ > σ(*F*^2^) is used only for calculating *R*-factors(gt) *etc*. and is not relevant to the choice of reflections for refinement. *R*-factors based on *F*^2^ are statistically about twice as large as those based on *F*, and *R*- factors based on ALL data will be even larger.

### Fractional atomic coordinates and isotropic or equivalent isotropic displacement parameters (Å^2^)

**Table:**

	*x*	*y*	*z*	*U*_iso_*/*U*_eq_	Occ. (<1)
N1	0.4885 (3)	0.37278 (15)	0.4386 (3)	0.0564 (8)	
H1	0.4232	0.4008	0.4606	0.068*	
N2	0.6055 (4)	0.39328 (15)	0.3787 (3)	0.0602 (8)	
N3	0.5568 (3)	0.08861 (13)	0.4727 (3)	0.0482 (7)	
H3	0.5116	0.0647	0.5330	0.058*	
O1	0.6492 (3)	−0.01035 (12)	0.3895 (3)	0.0661 (8)	
O2	0.7049 (18)	0.5320 (7)	0.454 (2)	0.074 (3)	0.66 (4)
H2	0.6727	0.4921	0.4459	0.111*	0.66 (4)
S1	0.9693 (10)	0.2364 (5)	0.5804 (11)	0.0641 (12)	0.692 (7)
O2'	0.660 (4)	0.5360 (13)	0.388 (4)	0.074 (6)	0.34 (4)
H2'	0.6242	0.4973	0.3696	0.111*	0.34 (4)
C12'	0.969 (8)	0.219 (3)	0.588 (9)	0.064 (17)	0.308 (7)
H12'	0.9461	0.2658	0.5743	0.077*	0.308 (7)
C1	0.6784 (4)	0.33555 (18)	0.3592 (4)	0.0523 (9)	
H1A	0.7642	0.3344	0.3190	0.063*	
C2	0.6089 (3)	0.27598 (15)	0.4074 (3)	0.0414 (8)	
C3	0.4862 (3)	0.30217 (17)	0.4600 (4)	0.0454 (8)	
C4	0.3889 (4)	0.25971 (18)	0.5179 (4)	0.0519 (9)	
H4	0.3085	0.2782	0.5529	0.062*	
C5	0.4155 (3)	0.18913 (17)	0.5218 (4)	0.0478 (9)	
H5	0.3522	0.1592	0.5609	0.057*	
C6	0.5368 (3)	0.16138 (16)	0.4678 (4)	0.0400 (8)	
C7	0.6358 (3)	0.20320 (16)	0.4120 (3)	0.0396 (8)	
C8	0.7669 (3)	0.17000 (16)	0.3616 (4)	0.0437 (8)	
H8	0.7972	0.1994	0.2809	0.052*	
C9	0.7228 (4)	0.09839 (17)	0.2912 (4)	0.0509 (9)	
H9A	0.6628	0.1052	0.1910	0.061*	
H9B	0.8090	0.0736	0.2744	0.061*	
C10	0.6422 (4)	0.05399 (18)	0.3891 (4)	0.0487 (9)	
C11	0.8917 (3)	0.16488 (19)	0.4956 (4)	0.0466 (8)	
C12	0.963 (4)	0.106 (2)	0.569 (4)	0.076 (10)	0.692 (7)
H12	0.9395	0.0605	0.5401	0.091*	0.692 (7)
S1'	0.962 (3)	0.0921 (16)	0.570 (3)	0.076 (3)	0.308 (7)
C13	1.0754 (5)	0.1238 (3)	0.6916 (6)	0.0920 (15)	
H13	1.1337	0.0921	0.7536	0.110*	
C14	1.0840 (4)	0.1937 (3)	0.7042 (5)	0.0813 (14)	
H14	1.1510	0.2159	0.7785	0.098*	
C15	0.749 (2)	0.5536 (8)	0.306 (3)	0.114 (5)	0.66 (4)
H15A	0.7454	0.5140	0.2359	0.137*	0.66 (4)
H15B	0.6848	0.5897	0.2562	0.137*	0.66 (4)
C16	0.896 (2)	0.5801 (13)	0.344 (3)	0.142 (6)	0.66 (4)
H16A	0.9040	0.6091	0.4347	0.213*	0.66 (4)
H16B	0.9190	0.6072	0.2589	0.213*	0.66 (4)
H16C	0.9621	0.5417	0.3639	0.213*	0.66 (4)
C15'	0.813 (5)	0.5334 (19)	0.378 (5)	0.114 (10)	0.34 (4)
H15C	0.8377	0.4902	0.3306	0.137*	0.34 (4)
H15D	0.8723	0.5385	0.4788	0.137*	0.34 (4)
C16'	0.827 (5)	0.595 (3)	0.276 (5)	0.142 (12)	0.34 (4)
H16D	0.7802	0.5855	0.1720	0.214*	0.34 (4)
H16E	0.9277	0.6047	0.2752	0.214*	0.34 (4)
H16F	0.7831	0.6354	0.3145	0.214*	0.34 (4)

### Atomic displacement parameters (Å^2^)

**Table:**

	*U*^11^	*U*^22^	*U*^33^	*U*^12^	*U*^13^	*U*^23^
N1	0.0542 (19)	0.0485 (18)	0.065 (2)	0.0099 (15)	0.0051 (16)	0.0007 (15)
N2	0.072 (2)	0.0467 (18)	0.062 (2)	0.0014 (16)	0.0107 (17)	0.0047 (14)
N3	0.0484 (16)	0.0392 (16)	0.0604 (18)	−0.0001 (13)	0.0187 (14)	0.0006 (13)
O1	0.0662 (17)	0.0407 (15)	0.097 (2)	−0.0024 (12)	0.0311 (15)	−0.0068 (13)
O2	0.087 (7)	0.059 (3)	0.085 (8)	−0.006 (4)	0.041 (6)	0.000 (5)
S1	0.0519 (16)	0.067 (3)	0.0727 (19)	−0.0077 (14)	0.0084 (13)	−0.0134 (15)
O2'	0.087 (14)	0.059 (7)	0.085 (15)	−0.006 (8)	0.041 (11)	0.000 (9)
C12'	0.052 (16)	0.07 (4)	0.073 (18)	−0.008 (19)	0.008 (12)	−0.01 (2)
C1	0.059 (2)	0.045 (2)	0.053 (2)	0.0024 (18)	0.0121 (17)	0.0042 (17)
C2	0.0448 (19)	0.0381 (19)	0.0393 (18)	−0.0010 (15)	0.0010 (15)	0.0022 (14)
C3	0.043 (2)	0.043 (2)	0.048 (2)	0.0020 (16)	−0.0002 (16)	0.0005 (16)
C4	0.0376 (19)	0.056 (2)	0.061 (2)	0.0068 (17)	0.0045 (17)	−0.0076 (18)
C5	0.0383 (19)	0.050 (2)	0.056 (2)	−0.0058 (15)	0.0100 (16)	−0.0019 (16)
C6	0.0370 (17)	0.0382 (18)	0.0446 (19)	0.0009 (15)	0.0057 (15)	0.0011 (15)
C7	0.0387 (18)	0.0425 (19)	0.0369 (18)	0.0004 (15)	0.0043 (14)	0.0013 (14)
C8	0.0468 (19)	0.0450 (19)	0.0421 (19)	0.0006 (16)	0.0159 (15)	0.0040 (15)
C9	0.051 (2)	0.056 (2)	0.047 (2)	0.0009 (17)	0.0125 (17)	−0.0040 (16)
C10	0.045 (2)	0.047 (2)	0.053 (2)	−0.0033 (17)	0.0078 (17)	−0.0086 (17)
C11	0.0355 (17)	0.061 (2)	0.047 (2)	0.0016 (18)	0.0153 (15)	0.0003 (18)
C12	0.075 (10)	0.061 (19)	0.087 (11)	0.008 (9)	−0.002 (7)	0.001 (9)
S1'	0.075 (5)	0.061 (7)	0.087 (6)	0.008 (3)	−0.002 (4)	0.001 (3)
C13	0.063 (3)	0.125 (5)	0.084 (4)	0.026 (3)	0.001 (3)	0.018 (3)
C14	0.048 (3)	0.127 (4)	0.069 (3)	−0.014 (3)	0.008 (2)	−0.022 (3)
C15	0.108 (11)	0.121 (9)	0.110 (12)	−0.022 (8)	0.009 (10)	0.038 (8)
C16	0.101 (13)	0.188 (16)	0.144 (15)	−0.013 (11)	0.041 (10)	0.031 (11)
C15'	0.11 (2)	0.121 (18)	0.111 (19)	−0.021 (17)	0.009 (18)	0.038 (17)
C16'	0.10 (3)	0.19 (3)	0.14 (3)	−0.01 (2)	0.04 (2)	0.03 (2)

### Geometric parameters (Å, º)

**Table:**

N1—N2	1.350 (4)	C7—C8	1.512 (4)
N1—C3	1.365 (4)	C8—C11	1.519 (4)
N1—H1	0.8600	C8—C9	1.531 (4)
N2—C1	1.325 (4)	C8—H8	0.9800
N3—C10	1.345 (4)	C9—C10	1.496 (5)
N3—C6	1.405 (4)	C9—H9A	0.9700
N3—H3	0.8600	C9—H9B	0.9700
O1—C10	1.233 (4)	C11—C12	1.41 (4)
O2—C15	1.48 (3)	C11—S1'	1.63 (3)
O2—H2	0.8200	C12—C13	1.41 (4)
S1—C14	1.618 (12)	C12—H12	0.9300
S1—C11	1.667 (10)	S1'—C13	1.50 (3)
O2'—C15'	1.45 (6)	C13—C14	1.343 (6)
O2'—H2'	0.8200	C13—H13	0.9300
C12'—C11	1.43 (6)	C14—H14	0.9300
C12'—C14	1.44 (7)	C15—C16	1.46 (4)
C12'—H12'	0.9300	C15—H15A	0.9700
C1—C2	1.412 (4)	C15—H15B	0.9700
C1—H1A	0.9300	C16—H16A	0.9600
C2—C3	1.400 (5)	C16—H16B	0.9600
C2—C7	1.415 (4)	C16—H16C	0.9600
C3—C4	1.379 (5)	C15'—C16'	1.51 (8)
C4—C5	1.373 (4)	C15'—H15C	0.9700
C4—H4	0.9300	C15'—H15D	0.9700
C5—C6	1.406 (4)	C16'—H16D	0.9600
C5—H5	0.9300	C16'—H16E	0.9600
C6—C7	1.375 (4)	C16'—H16F	0.9600
			
N2—N1—C3	111.9 (3)	H9A—C9—H9B	107.6
N2—N1—H1	124.1	O1—C10—N3	121.8 (3)
C3—N1—H1	124.1	O1—C10—C9	122.5 (3)
C1—N2—N1	106.1 (3)	N3—C10—C9	115.7 (3)
C10—N3—C6	124.1 (3)	C12—C11—C12'	99 (3)
C10—N3—H3	118.0	C12—C11—C8	130.9 (17)
C6—N3—H3	118.0	C12'—C11—C8	130 (3)
C15—O2—H2	109.5	C12'—C11—S1'	105 (3)
C14—S1—C11	94.4 (5)	C8—C11—S1'	125.1 (10)
C15'—O2'—H2'	109.5	C12—C11—S1	108.0 (18)
C11—C12'—C14	114 (4)	C8—C11—S1	121.1 (4)
C11—C12'—H12'	122.8	S1'—C11—S1	113.8 (10)
C14—C12'—H12'	122.8	C11—C12—C13	113 (3)
N2—C1—C2	111.2 (3)	C11—C12—H12	123.3
N2—C1—H1A	124.4	C13—C12—H12	123.3
C2—C1—H1A	124.4	C13—S1'—C11	97.5 (17)
C3—C2—C1	104.7 (3)	C14—C13—C12	109.0 (16)
C3—C2—C7	119.7 (3)	C14—C13—S1'	119.1 (12)
C1—C2—C7	135.6 (3)	C14—C13—H13	125.5
N1—C3—C4	131.3 (3)	C12—C13—H13	125.5
N1—C3—C2	106.1 (3)	S1'—C13—H13	115.4
C4—C3—C2	122.6 (3)	C13—C14—C12'	104 (2)
C5—C4—C3	117.4 (3)	C13—C14—S1	115.1 (5)
C5—C4—H4	121.3	C13—C14—H14	122.4
C3—C4—H4	121.3	C12'—C14—H14	133.3
C4—C5—C6	121.1 (3)	S1—C14—H14	122.4
C4—C5—H5	119.4	C16—C15—O2	106 (3)
C6—C5—H5	119.4	C16—C15—H15A	110.4
C7—C6—N3	119.6 (3)	O2—C15—H15A	110.4
C7—C6—C5	122.1 (3)	C16—C15—H15B	110.4
N3—C6—C5	118.4 (3)	O2—C15—H15B	110.4
C6—C7—C2	117.1 (3)	H15A—C15—H15B	108.6
C6—C7—C8	119.2 (3)	O2'—C15'—C16'	101 (6)
C2—C7—C8	123.7 (3)	O2'—C15'—H15C	111.5
C7—C8—C11	111.3 (3)	C16'—C15'—H15C	111.5
C7—C8—C9	108.3 (3)	O2'—C15'—H15D	111.5
C11—C8—C9	112.1 (3)	C16'—C15'—H15D	111.5
C7—C8—H8	108.4	H15C—C15'—H15D	109.3
C11—C8—H8	108.4	C15'—C16'—H16D	109.5
C9—C8—H8	108.4	C15'—C16'—H16E	109.5
C10—C9—C8	114.0 (3)	H16D—C16'—H16E	109.5
C10—C9—H9A	108.8	C15'—C16'—H16F	109.5
C8—C9—H9A	108.8	H16D—C16'—H16F	109.5
C10—C9—H9B	108.8	H16E—C16'—H16F	109.5
C8—C9—H9B	108.8		
			
C3—N1—N2—C1	0.9 (4)	C14—C12'—C11—C8	−179 (2)
N1—N2—C1—C2	−0.2 (4)	C14—C12'—C11—S1'	4 (5)
N2—C1—C2—C3	−0.4 (4)	C14—C12'—C11—S1	−163 (25)
N2—C1—C2—C7	179.4 (3)	C7—C8—C11—C12	116 (2)
N2—N1—C3—C4	179.6 (3)	C9—C8—C11—C12	−5 (2)
N2—N1—C3—C2	−1.2 (3)	C7—C8—C11—C12'	−61 (4)
C1—C2—C3—N1	0.9 (3)	C9—C8—C11—C12'	177 (4)
C7—C2—C3—N1	−178.9 (3)	C7—C8—C11—S1'	115.6 (12)
C1—C2—C3—C4	−179.8 (3)	C9—C8—C11—S1'	−5.9 (12)
C7—C2—C3—C4	0.3 (5)	C7—C8—C11—S1	−64.4 (5)
N1—C3—C4—C5	178.6 (3)	C9—C8—C11—S1	174.2 (5)
C2—C3—C4—C5	−0.5 (5)	C14—S1—C11—C12	−1.0 (18)
C3—C4—C5—C6	−0.4 (5)	C14—S1—C11—C12'	14 (20)
C10—N3—C6—C7	−19.3 (4)	C14—S1—C11—C8	179.7 (3)
C10—N3—C6—C5	161.2 (3)	C14—S1—C11—S1'	−0.3 (12)
C4—C5—C6—C7	1.5 (5)	C12'—C11—C12—C13	−1 (4)
C4—C5—C6—N3	−179.0 (3)	C8—C11—C12—C13	−179.7 (11)
N3—C6—C7—C2	179.0 (3)	S1'—C11—C12—C13	−173 (28)
C5—C6—C7—C2	−1.5 (4)	S1—C11—C12—C13	1 (3)
N3—C6—C7—C8	−1.9 (4)	C12—C11—S1'—C13	7 (24)
C5—C6—C7—C8	177.6 (3)	C12'—C11—S1'—C13	−3 (3)
C3—C2—C7—C6	0.6 (4)	C8—C11—S1'—C13	179.8 (6)
C1—C2—C7—C6	−179.2 (3)	S1—C11—S1'—C13	−0.2 (17)
C3—C2—C7—C8	−178.5 (3)	C11—C12—C13—C14	−1 (3)
C1—C2—C7—C8	1.7 (5)	C11—C12—C13—S1'	175 (17)
C6—C7—C8—C11	−89.4 (3)	C11—S1'—C13—C14	0.8 (17)
C2—C7—C8—C11	89.7 (3)	C11—S1'—C13—C12	−4 (14)
C6—C7—C8—C9	34.2 (4)	C12—C13—C14—C12'	2 (4)
C2—C7—C8—C9	−146.7 (3)	S1'—C13—C14—C12'	1 (3)
C7—C8—C9—C10	−49.2 (4)	C12—C13—C14—S1	−0.3 (18)
C11—C8—C9—C10	74.0 (4)	S1'—C13—C14—S1	−1.1 (14)
C6—N3—C10—O1	−175.2 (3)	C11—C12'—C14—C13	−3 (5)
C6—N3—C10—C9	2.4 (4)	C11—C12'—C14—S1	165 (21)
C8—C9—C10—O1	−149.4 (3)	C11—S1—C14—C13	0.8 (7)
C8—C9—C10—N3	33.0 (4)	C11—S1—C14—C12'	−12 (17)
C14—C12'—C11—C12	3 (5)		

### Hydrogen-bond geometry (Å, º)

**Table:**

*D*—H···*A*	*D*—H	H···*A*	*D*···*A*	*D*—H···*A*
N1—H1···O2^i^	0.86	1.99	2.838 (16)	170
N3—H3···O1^ii^	0.86	2.04	2.863 (4)	160
O2—H2···N2	0.82	2.05	2.855 (14)	167
C8—H8···S1^iii^	0.98	2.86	3.802 (6)	162
C9—H9*A*···N1^iii^	0.97	2.56	3.529 (7)	175

Symmetry codes: (i) −*x*+1, −*y*+1, −*z*+1; (ii) −*x*+1, −*y*, −*z*+1; (iii) *x*, −*y*+1/2, *z*−1/2.
